# Prevalence of Malaria and COVID-19 Infection in Akure North Local Government Area of Ondo State, Nigeria

**DOI:** 10.1155/2023/9529563

**Published:** 2023-01-05

**Authors:** Iyabo Adepeju Simon-Oke, Oluwaseun Bunmi Awosolu, Olatunji Odeyemi

**Affiliations:** Department of Biology, Federal University of Technology, Akure, Nigeria

## Abstract

**Introduction:**

The prevalence of malaria and coronavirus disease (COVID-19) is highly devastating and has led to a serious public health challenge worldwide. In order to ensure proper control and elimination, the State Ministry of Health (MoH) Ondo State, Nigeria conducted medical examinations in Iju/Itaogbolu, Igoba, and Ogbese Health centers to identify and confirm cases of COVID-19 and malaria infection. This study provides the outcome of the epidemiological investigation of the prevalence of COVID-19 and malaria in Akure North Local Government Area of Ondo State, Nigeria.

**Method:**

The study was a hospital-based secondary data analysis comprising of 11,389 and 682 individuals who visited various health centers in Akure North Local Government Area (LGA) for medical examinations on malaria and COVID-19, respectively. The COVID-19 cases were investigated using the fluid sample collected with a nasal swab or a throat swab, or spit of saliva into a tube and confirmed by real-time polymerase chain reaction (RT-PCR). The *Plasmodium falciparum* histidine-rich protein 2 (PfHRP2) RDT was employed to detect the *P.falciparum* antigen among participants' blood samples.

**Results:**

The total prevalence of malaria and COVID-19 were 67.6% and 12.4%, respectively. Meanwhile, the month of September recorded the highest malaria prevalence of 81.8% while the month of April recorded the least malaria prevalence of 56.4%. Similarly, the highest case of COVID-19 (18.8%) was recorded in the month of November while the least case (2.4%) was recorded in the month of April (*p* < 0.05). The age range of 12-59 months had the highest malaria prevalence of 74.9% while 0-24 days age range recorded the least prevalence of 15.2% (*p* > 0.05). Apparently, the children were more infected with malaria parasites while adults were more infected with COVID-19.

**Conclusion:**

Conclusively, malaria and COVID-19 infections were prevalent in the study area. Thus, the people should be enlightened on the deadly risk of malaria and COVID-19 through the health workers, social media, and the community leaders to ensure compliance with appropriate preventive measures.

## 1. Introduction

Malaria and coronavirus disease (COVID-19) are serious infectious disease of major public health challenge, leading to untold morbidity and mortality worldwide, particularly in African countries [1]. The coronavirus disease (COVID-19) is a highly infectious and pathogenic viral infection caused by severe acute respiratory syndrome coronavirus 2 (SARS-CoV-2). It resulted to a global pandemic that led to a dramatic loss of human lives worldwide [2]. Coronavirus is one of the major pathogens that primarily target the human respiratory system. Previous outbreaks of coronaviruses (CoVs) include the severe acute respiratory syndrome (SARS)-CoV and the Middle East respiratory syndrome coronavirus (MERS-CoV) which have been previously characterized as agents that are a great public health threat. Coronaviruses have been classified to belong to the Coronaviridae family in the Nidovirales order [3]. According to the World Health Organization, the 2003 SARS outbreak originated from civet cats and the 2012 MERS outbreak from dromedary camels.

These viruses were thought to infect only animals until the world witnessed a severe acute respiratory syndrome (SARS) outbreak caused by SARS-CoV, 2002 in Guangdong, China [4]. About a decade thereafter, another pathogenic coronavirus, known as Middle East respiratory syndrome coronavirus (MERS-CoV) caused an endemic in Middle Eastern countries [5] Recently, at the end of 2019, Wuhan an emerging business hub of China experienced an outbreak of a novel coronavirus which led to the death of thousands of individuals within the first fifty days of the epidemic [6]. The disease has spread to more than 200 countries with over 200 million cases, approximately 4.1 million deaths, and 180 million recoveries worldwide according to the World Health Organization as of August 2021 [7]. In Nigeria, 180,661 cases were confirmed, 165,122 cases were discharged, and 2163 deaths were recorded in 36 states including the Federal Capital Territory as of 8^th^ August 2021 [8].

Similarly, for many decades, malaria remains a serious public health challenge in the world, especially in the tropical and subtropical regions. Major *Plasmodium* species include *Plasmodium falciparum*, *Plasmodium vivax*, *Plasmodium ovale*, and *Plasmodium malariae* out of which *P. falciparum* has been reported to be the deadliest and therefore accounting for about 95% of all malaria death in African countries [1]. Currently, despite the reduction in malaria cases in the past 15 years, millions of people, particularly in the African continent still battle with malaria due to poor socioeconomic status and lack of preventive and treatment tools [9]. There were an estimated 228 million alarming cases and 405,000 deaths due to malaria in 2018 [10]. Similarly, Nigeria accounted for about 25% of all malaria cases with 24% mortality worldwide in 2018, thereby bearing the highest burden of malaria infection globally [10]. This has led to increased level of poverty as a result of unexpected expenses on treatment, control and prevention. Moreover, time expected to be at work and school is wasted on ill-health due to malaria infection thereby further aggravating the poor conditions, both in rural and urban areas [11].

In Nigeria, prevalence of malaria infection varies in different geopolitical zones of the country as a result of varying environmental and seasonal conditions which influence the breeding habit and abundance of mosquito vectors. Despite several control strategies employed by individuals and government to reduce the prevalence of malaria, there are still reports of high prevalence in the country, especially in the southwestern region due to high annual rainfall and moderate temperature [12]. Therefore, this study was carried out to determine the prevalence of malaria and COVID-19 in Akure North Local Government of Ondo State, Nigeria.

## 2. Materials and Methods

### 2.1. Study Area

**Figure 1:**
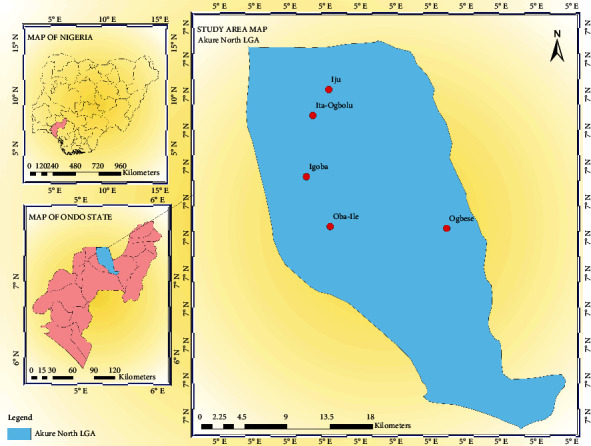
Map of Akure North Local Government Area of Ondo State, Nigeria.

The study was conducted in Akure North Local Government Area in Ondo State, Nigeria. The Local Government Area comprises of five major communities which include Iju, Itaogbolu, Oba Ile, Igoba, and Ogbese (Figure 1). These communities are located between latitudes 5°45′ and 7°52′N and longitudes 4°20′ and 6°05′E. The population of the area is approximately 198,000 and it has an area of 660 km^2^ (250 sq. mi). The vegetation type of Akure North Local Government Area is typically rainforest dominated by abundant trees and grasses. The predominant occupations in the communities are farming and trading.

### 2.2. Sample Size Determination

The sample size was determined using of Raosoft sample size calculator at 5% margin of error and 95% confidence interval. The sample size obtained for the study was based on the population size (198,000) of the LGA. The sample size generated was 384. In spite the sample size generated, 682 and 11,389 individuals were examined for COVID-19 and malaria infections, respectively.

### 2.3. Study Design and Population

This was a hospital-based secondary data analysis study. The study included 11,389 and 682 individuals who visited the health center for medical examinations on malaria and COVID-19, respectively. The health centers were Iju/Itaogbolu, Igoba, and Ogbese health centers. The study was carried out at each of the major communities of Akure North Local Government Area between January 2020 and December 2020. All individuals visiting the health centers and having symptom of fever with temperature ≥37.5°C were included in the study. However, patients who had treated malaria three weeks before the study were excluded.

### 2.4. Sample Collection and Examination

Using the COVID-19 operational case definitions developed by the Nigeria Centre for Disease Control, active case search visits/case finding was conducted in the communities and health facilities across the five health centers of the Local Government Areas (LGAs) by the State RRT in order to identify suspected cases. The COVID-19 cases were investigated using the fluid sample collected with a nasal swab or a throat swab, or spit of saliva into a tube to produce a saliva sample. Samples collected were transported under reverse cold chain temperatures (+2°C–+8°C) to designated national reference laboratory for confirmatory testing for COVID-19 by real-time polymerase chain reaction (RT-PCR).

### 2.5. Rapid Diagnostic Test Analysis

The rapid malaria test, PfHRP2 CareStart™ (Access BIO, Inc., Monmouth Junction, New Jersey, USA) was used to detect *P. falciparum* in participants' blood samples. About 5 *μ*l of blood sample was collected using a micropipette provided. The RDT was based on lateral flow immune chromatography in cassette format which can detect *P. falciparum* histidine-rich protein 2 (PfHRP2). The test was carried out on the field and read within 20 min according to the manufacturer's instructions.

### 2.6. Data Analysis

All data collected were recorded on Microsoft Excel Sheet and then exported to Statistical Package for Social Sciences (SPSS) version 26 (IBM SPSS Statistics) where the data were analyzed. Test for significance was done using Pearson's Chi-Square Tests at *p* < 0.05. All charts were created using Microsoft Excel.

## 3. Results

Table 1 shows the prevalence of COVID-19 according to month. The overall COVID-19 cases reported was 85 (12.46%). Apparently, the month of November recorded the highest number of cases [16 (18.82%)] with a significant difference (*p* < 0.05). The least confirmed case was recorded in the month of April [2 (2.4%)] with no significant difference. Similarly, Table 2 revealed that the overall total prevalence of malaria infection for the year was 68.6%. The month of September recorded the highest prevalence of malaria infection (81.8%) while the month of April recorded the least prevalence of malaria infection (56.4%). The age range of 12-59 months had the highest malaria prevalence of 74.92% while 0-24 days recorded the least malaria prevalence of 15.24%. However, there was no significant difference among the age groups (*p* > 0.05) as shown in Table 3.

**Figure 2:**
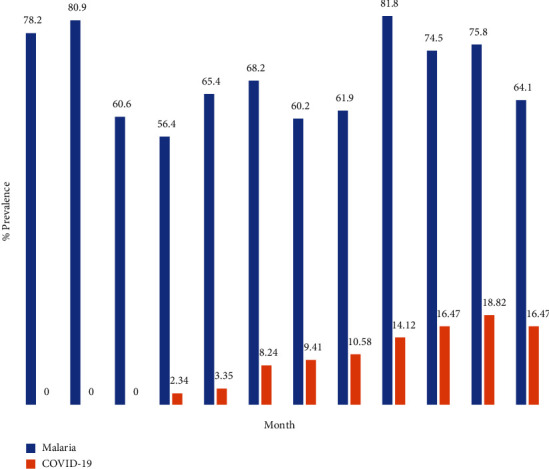
Percentage infection of malaria and COVID-19 in Akure North Local Government Area of Ondo State, Nigeria.

Figure 2 shows that in the month of January to March, there was no record for COVID-19 infection but there was high prevalence of malaria infection (80.9%) for the three months. For COVID-19 infection, the highest number of cases (18.82%) was obtained in the month of November while for malaria, the highest prevalence of 81.87% was recorded in September. The least prevalence for both COVID-19 and malaria (2.24% vs. 56.4%) were obtained in the month of April. There was a significant difference in the prevalence of COVID-19 and malaria disease (*p* < 0.05).

**Figure 3:**
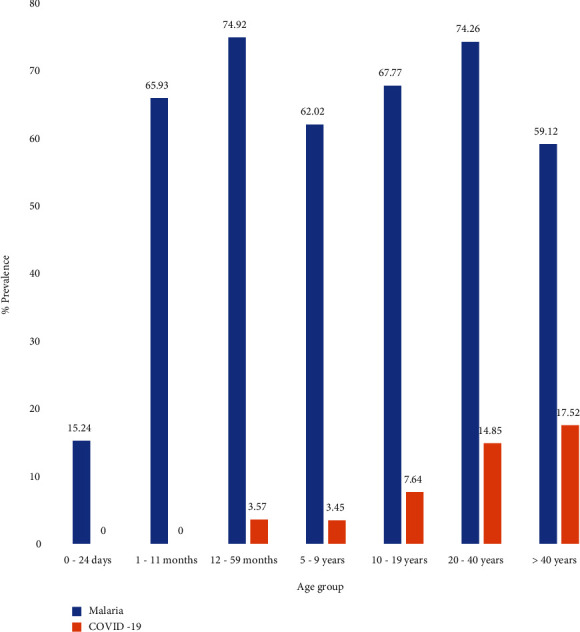
Percentage infection of malaria and COVID-19 in relation to the age group in Akure North Local Government Area of Ondo State, Nigeria.

Additionally, the age group >40 years recorded the highest prevalence of 17.82% for COVID-19 infection while the least prevalence of 3.45% was recorded among age group 5-9 years. As for malaria infection, the age range 12-59 months recorded the highest prevalence of 74.92% while 0-24 days recorded a malaria prevalence of 15.24%. There was a significant difference (*p* < 0.05) in the prevalence of COVID-19 and malaria infection in relation to age groups (Figure 3).

Prevalence of malaria infection among patients in the different study locations within the LGA is presented in Table 4. It was observed that the four towns presented high prevalence of malaria infection with the highest prevalence recorded in Ogbese town (88.4%) while the least prevalence was recorded in Igoba town (59.2%). Statistical analysis revealed that there was no significant difference in the prevalence of malaria infection in the different locations (*p* > 0.05).

## 4. Discussion

The findings in this research revealed that a high proportion of the confirmed COVID-19 cases were found in males than the females. This is consistent with previous studies in Oyo State, Nigeria [13] and Wuhan, China [14]. The high proportion of COVID-19 infection among the males could be attributed to the fact that males are economically active group, suggesting a potential role of socio-economic or work-related activities rather than immunological capacity. Men are more likely to engage in economic activities outside of the household and potentially become more exposed to SARS-CoV-2 infection than women [15]. Additional factor responsible could be the poor male compliance and attitude towards COVID-19 preventive measures and protocol such as frequent hand washing, wearing of face mask, and defiant to stay at home orders among men compare to women as previously reported by Bwire [16] and Isere et al. [17]. The age group >40 years recorded the highest prevalence of COVID-19 infection while the least prevalence of COVID-19 infection was recorded among 5-9 years old. This could be as a result of the lack or little exposure of this age group 5-9 years old to person-to-person human interactions due to daily commercial activities while >40 years old were more likely infected due to unrestricted person-to-person contacts. Other factors such as crowded conditions and clustered settlement pattern of the houses could have facilitated the spread of the infection [1, 18].

The highest cases of COVID-19 infection were obtained in the month of November while the least was recorded in the month of April. This may likely be as a result of reasonable improvement in the active contact tracing and marked increase in testing rate of suspected individuals, thereby leading to high number of individuals infected with COVID-19 being detected. However, least number of people was likely infected in April since the COVID-19 infection was newly spreading in the study area with few people being diagnosed. Similarly, it could be due to the onset of rainfall and the resulting low temperature which may likely lead to reduced social interraction. Also, indoor relative humidity becomes reduced during the dry season (November to March) and higher in the rainy season (April to October) while the outdoor relative humidity is vice versa. The humidity of the environment during the dry season may encourage viral transmission [19]. Relative humidity which is strongly linked to temperature is a better predictor of virus survival. Other environmental variables such as temperature, sunlight and UV radiation might also contribute to the seasonal transmission of Covid-19 by influencing the inactivation of virus in air and on surfaces [20].

Regarding malaria infection, the month of September recorded the highest malaria prevalence unlike COVID-19 infection which recorded the highest in month of November. Previous reports revealed that malaria infection thrives more during the rainy season due to availability of mosquito breeding habitats and the resulting increase in mosquito population. Temperature, rainfall, and humidity have been widely associated with the dynamics of malaria vector population and therefore with the spread of the disease. The temporal change in mosquito abundance is mainly caused by rainfall. The number of larval habitats was substantially higher in the rainy season than in the dry season. Apart from the importance of environmental and ecological factors such as breeding sites, humidity, temperature, and rainfall, human activities such as agricultural practices and lumbering also contribute immensely to the distribution and abundance of these mosquito vectors, especially the availability of host for blood meal [21].

Our findings revealed that those who belong to age group 12-59 months old were more infected with malaria parasites than 0-24 days and this could be as a result of lack of immunity and constant exposure to the mosquito vectors. Apparently, 0-24 days old were more protected against mosquito vectors than 12-59 months due to mother care through constant wearing of protective clothing and probably sleeping under insecticide treated nets. Similarly, protective immunity from mother to child could play a major role in protection against malaria infection. In comparison to COVID-19 infection, age group >40 recorded the highest prevalence while the least was observed in 12-59 months old. Age group 12-59 months old recorded different prevalence for malaria and COVID-19 (74.92% vs. 3.57%). This variation could likely be attributed to the different modes of transmission and levels of control intervention. While malaria infection was a previously endemic disease in the study areas, COVID-19 infection was relatively new though it was spreading fast at an alarming rate through person-to-person transmission and human interactions arising from day-to-day commercial activities of individuals [15–18]. Variation in prevalence of malaria parasite and COVID-19 among the children sampled could be attributed in part to the difference in malaria transmission pattern, season, and the use of malaria prevention tools [22]. Also, presence of naturally acquired immunity in children from their mother could be responsible for the malaria prevalence pattern recorded among the children in this study.

High prevalence of malaria infection recorded in each of the studied town in the LGA is alarming which calls for serious public health intervention. However, several studies have revealed variation in the prevalence of malaria infection based on different sampling locations [23–25], and our findings in this study is not an exception as there were variations in the prevalence of malaria infection in the different studied towns within the LGA. Several factors such as human activities, vegetation, environmental conditions, vectorial capacity of malaria vectors, and the different malaria control strategies employed in the different location could have brought about these variations [25].

## 5. Conclusion

The outcome of this research work indicates that there is high transmission of coronavirus disease (COVID-19) and malaria infections in the study area. The children were more infected with malaria parasites while for COVID-19, the adults were more infected. The high cases of both malaria and COVID-19 among the affected age groups require public health intervention. Thus, public enlightenment is recommended in form of intensified risk communication through health workers, social media, enhanced surveillance activities, and the use of community structures such as community, religious, and market leaders to ensure compliance with public health COVID-19 preventive measures, particularly in the urban areas. Furthermore, there is a need to prioritize public health interventions including training and vaccination among the vulnerable groups, including the health care workers.

## Figures and Tables

**Table 1:** Prevalence of COVID-19 according to month in Akure North Local Government Area of Ondo State, Nigeria.

Month	Suspected		Confirmed		Total suspected	Total confirmed
	Male (%)	Female (%)	Male (%)	Female (%)		
Apr.	10 (3.4)	9 (2.3)	1 (2.2)	1 (2.6)	19 (2.8)	2 (2.4)
May	29 (9.8)	38 (9.8)	1 (2.2)	2 (5.1)	67 (9.8)	3 (3.5)
Jun.	41 (13.9)	52 (13.5)	4 (8.7)	3 (7.7)	93 (13.6)	7 (8.2)
Jul.	43 (14.5)	31 (8.0)	5 (10.9)	3 (7.7)	74 (10.9)	8 (9.4)
Aug.	18 (6.1)	39 (10.1)	5 (10.9)	4 (10.3)	57 (8.4)	9 (10.6)
Sept.	21 (7.1)	42 (10.9)	7 (15.2)	5 (12.8)	63 (9.2)	12 (14.1)
Oct.	24 (8.1)	48 (12.4)	8 (17.4)	6 (15.4)	72 (10.6)	14 (16.5)
Nov.	49 (16.5)	67 (17.4)	7 (15.2)	9 (23.1)	116 (17.0)	16 (18.8)
Dec.	61 (20.6)	60 (15.5)	8 (17.4)	6 (15.4)	121(17.7)	14 (16.5)
Total	296 (100.0)	386 (100.0)	46 (100)	39 (100.0)	682 (100.0)	85 (100.0)

**Table 2:** Prevalence of malaria infection in Akure North Local Government Area of Ondo State, Nigeria.

Month	Number examined	Number positive	Prevalence (%)
January	797	623	78.2
February	817	661	80.9
March	744	451	60.6
April	1107	624	56.4
May	980	641	65.4
June	642	438	68.2
July	1039	625	60.2
August	670	600	61.9
September	716	586	81.8
October	988	736	74.5
November	1436	1089	75.8
December	1162	745	60.4
Total	11398	7819	68.6

*χ* ^2^ = 132.00; df = 11; *p* = 0.233 (*p* > 0.05).

**Table 3:** Prevalence of malaria infection in relation to age in Akure North Local Government Area of Ondo State, Nigeria.

Age range	Number examined	Number positive	Prevalence (%)
0–24 days	105	16	15.24
1–11 months	992	654	65.93
12–59 months	2197	1646	74.92
5–9 years	1743	1081	62.02
10–19 years	1778	1205	67.77
20–40 years	2626	1950	74.26
>40 years	1957	1157	59.12
Total	11398	7709	67.64

Chi-square (*χ*^2^) = 42.00; df = 6; *p* value = 0.227; Phi = 0.227; Cramer′s V = 0.227.

**Table 4:** Prevalence of malaria infection in different towns in North LGA of Ondo State, Nigeria.

Study site	Suspected cases (%)	Confirmed cases (%)
Iju/Itaogbolu PHC	3523 (30.9)	2329 (66.1)
Igoba PHC	2404 (21.1)	1422 (59.2)
Oba Ile PHC	3075 (27.0)	1951 (63.4)
Ogbese PHC	2396 (21.0)	2117 (88.4)
Total	11398 (100.0)	7819 (68.6)

Chi-square (*χ*^2^) = 83.25; df = 3; *p* value = 0.131; Phi = 0.131; Cramer′s V = 0.131.

## Data Availability

Additional data can be made available from the corresponding author upon reasonable request.
